# General Information Regarding Examination for License to Practice Dentistry in California

**Published:** 1923-05

**Authors:** C. A. Herrick

**Affiliations:** Secretary, 133 Geary Street, San Francisco, Cal.


### GENERAL INFORMATION REGARDING EXAMINATION FOR LICENSE TO PRACTICE DENTISTRY IN CALIFORNIA

All persons desiring to engage in the practice of dentistry in California must obtain a license by passing a satisfactory examination before the Board of Dental Examiners.

The law of the state makes no provision for the issuance of temporary permits to practice or the interchange of licenses with other states.

Section six of the dental law determines the eligibility of applicants to the examination and reads in part as follows:

SEC. 6. Any person over twenty-one years of age shall be eligible to take an examination before the board of dental examiners of California, upon making application therefor and upon (1) paying a fee of twenty-five dollars; (2) furnishing satisfactory testimonials of good moral character; and (3) furnishing satisfactory evidence of having graduated from a reputable dental college, which must have been approved by the board of dental examiners of California; *provided,* that after August 1, 1918, he shall also file his diploma or certificate of graduation with recommendations from a high school accredited to the University of California or any other university of equal standing; or a certificate signed by a state superintendent of public instruction, or similar officer, to the effect that such applicant has had scholastic preparation equivalent in all respects to that demanded for graduation with recommendations from a high school giving a four-year course of instruction in the state from which such certificate is issued; (4) in lieu of such diploma or certificate from an accredited high school, such applicant, after said date, may and with like effect furnish to said board of dental examiners a certificate from the board of dental examiners, or similar official body, of some other state in the United States, showing that such applicant has been a duly licensed practitioner of dentistry in such other state for a period of at least five years.

All applications for examination must be made out and filed with the Board on the day set for the beginning of examination and each application must be accompanied by the fee of $25.00. Each applicant shall file with the secretary of the Board at least 15 days prior to the date selected for beginning the examination, the following credentials: (1) diploma or certificate from a reputable dental college approved by the Board of Dental Examiners of California; (2) a diploma from an accredited high school or a certificate signed by a State Superintendent of Public Instruction (or similar officer) to the effect that such applicant has had scholastic preparation equivalent in all respects to that demanded for graduation from a high school giving a four-year course of instruction in the state from which such certificate is issued (a certificate signed by a deputy superintendent or an examiner for entrance to a dental college or other school will not be accepted by the Board); (3) a satisfactory testimonial of good moral character; (4) a recent unmounted photograph of the applicant.

Testimonials of good moral character for applicants who are licensed in some other state of the United States, should bear the signature and seal of the Secretary of the Board of Dental Examiners, or the Secretary of the State Dental Association of that state.

An applicant for examination, who is licensed in some other state of the United States, must present to the Board his or her license; or in lieu of said license a certified copy of the records of a Board of Dental Examiners or County Clerk showing that he or she is licensed in some other state.

The theoretical examination shall include, written in the English language, questions on the following subjects: Anatomy, histology, physiology, anæsthesia, materia medica, pathology, bacteriology, therapeutics, oral surgery, chemistry, metallurgy, operative dentistry, prosthetic dentistry and orthodontia.

The requirements for the practical examination in operative and prosthetic dentistry shall be decided upon and announced to the applicants on the day selected for beginning the theoretical examination.

All dental engines, instruments, materials and patients must be furnished by the applicant. A chair will be furnished by the Board.

Each applicant must be supplied with a fountain pen.

Three (3) examinations are held each year—two during the summer and one during the winter.

Those asking for information should send a stamp for reply.

The next examination will be held in (date not fixed, probably be May or June). For information regarding next examination write the secretary.

By order of the Board,

C. A. Herrick, D. D. S., *Secretary*,

133 Geary Street, San Francisco, Cal.

